# Association of Medicaid Expansion Under the Affordable Care Act With Insurance Status, Cancer Stage, and Timely Treatment Among Patients With Breast, Colon, and Lung Cancer

**DOI:** 10.1001/jamanetworkopen.2019.21653

**Published:** 2020-02-19

**Authors:** Samuel U. Takvorian, Arman Oganisian, Ronac Mamtani, Nandita Mitra, Lawrence N. Shulman, Justin E. Bekelman, Rachel M. Werner

**Affiliations:** 1 Division of Hematology and Oncology, Perelman School of Medicine, University of Pennsylvania, Philadelphia; 2 Penn Center for Cancer Care Innovation, Abramson Cancer Center, University of Pennsylvania, Philadelphia; 3 Leonard Davis Institute of Health Economics, University of Pennsylvania, Philadelphia; 4 Department of Biostatistics, Epidemiology and Informatics, Perelman School of Medicine, University of Pennsylvania, Philadelphia; 5 Department of Radiation Oncology, Perelman School of Medicine, University of Pennsylvania, Philadelphia; 6 Department of Medical Ethics and Health Policy, Perelman School of Medicine, University of Pennsylvania, Philadelphia; 7 Division of General Internal Medicine, Perelman School of Medicine, University of Pennsylvania, Philadelphia; 8 Center for Health Equity Research and Promotion, Corporal Michael J. Crescenz VA Medical Center, Philadelphia, Pennsylvania

## Abstract

**Importance:**

The effect of the Patient Protection and Affordable Care Act’s Medicaid expansion on cancer care delivery and outcomes is unknown. Patients with cancer are a high-risk group for whom treatment delays are particularly detrimental.

**Objective:**

To examine the association between Medicaid expansion and changes in insurance status, stage at diagnosis, and timely treatment among patients with incident breast, colon, and non–small cell lung cancer.

**Design, Setting, and Participants:**

This quasi-experimental, difference-in-differences (DID) cross-sectional study included nonelderly adults (aged 40-64 years) with a new diagnosis of invasive breast, colon, or non–small cell lung cancer from January 1, 2011, to December 31, 2016, in the National Cancer Database, a hospital-based registry capturing more than 70% of incident cancer diagnoses in the United States. Data were analyzed from March 8 to August 15, 2019.

**Exposures:**

Residence in a state that expanded Medicaid on January 1, 2014.

**Main Outcomes and Measures:**

The primary outcomes were insurance status, cancer stage, and timely treatment within 30 and 90 days of diagnosis.

**Results:**

A total of 925 543 patients (78.6% women; mean [SD] age, 55.0 [6.5] years; 14.2% black; and 5.7% Hispanic) had a new diagnosis of invasive breast (58.9%), colon (14.6%), or non–small cell lung (26.5%) cancer; 48.3% resided in Medicaid expansion states and 51.7% resided in nonexpansion states. Compared with nonexpansion states, the percentage of uninsured patients decreased more in expansion states (adjusted DID, −0.7 [95% CI, −1.2 to −0.3] percentage points), and the percentage of early-stage cancer diagnoses rose more in expansion states (adjusted DID, 0.8 [95% CI, 0.3 to 1.2] percentage points). Among the 848 329 patients who underwent cancer-directed therapy within 365 days of diagnosis, the percentage treated within 30 days declined from 52.7% before to 48.0% after expansion in expansion states (difference, −4.7 [95% CI, −5.1 to −4.5] percentage points). In nonexpansion states, this percentage declined from 56.9% to 51.5% (difference, −5.4 [95% CI, −5.6 to −5.1] percentage points), yielding no statistically significant DID in timely treatment associated with Medicaid expansion (adjusted DID, 0.6 [95% CI, −0.2 to 1.4] percentage points).

**Conclusions and Relevance:**

This study found that, among patients with incident breast, colon, and lung cancer, Medicaid expansion was associated with a decreased rate of uninsured patients and increased rate of early-stage cancer diagnosis; no evidence of improvement or decrement in the rate of timely treatment was found. Further research is warranted to understand Medicaid expansion’s effect on the treatment patterns and health outcomes of patients with cancer.

## Key Points

**Question:**

Three years after implementation of the Patient Protection and Affordable Care Act, was Medicaid expansion associated with changes in insurance status and cancer stage at diagnosis without delaying time to treatment?

**Findings:**

In this cross-sectional study of 925 543 patients with incident breast, colon, and lung cancer, Medicaid expansion was associated with a decreased rate of uninsured patients and an increased rate of early-stage cancer diagnosis; it was not associated with changes in the rate of timely treatment.

**Meaning:**

Among patients with newly diagnosed breast, colon, and lung cancer, Medicaid expansion was associated with increased insurance coverage and earlier-stage cancer diagnosis without evidence of treatment delay.

## Teaser

This cross-sectional study examines the association between Medicaid expansion following the Patient Protection and Affordable Care Act (ACA) and changes in insurance status, cancer stage at diagnosis, and timely treatment among patients with incident breast, colon, and non–small cell lung cancer.

## Introduction

The Patient Protection and Affordable Care Act (ACA) expanded Medicaid eligibility in participating states to nonelderly adults with incomes at or below 138% of the federal poverty level. Since its passage, more than 20 million US residents have gained insurance coverage.^1^ These expansions have been associated with improved access to care, affordability, and, for certain surgical procedures and medical conditions, health outcomes.^2,3,4,5,6,7,8^ However, studies have also suggested unintended consequences, such as lengthened wait times,^2,6^ and debate about the overall effect of the expansions at the state and federal levels is ongoing.^9^

In cancer care, preliminary studies have shown Medicaid expansion to be associated with reduced rates of uninsured patients,^10,11,12^ increased screening,^13,14^ and earlier stage at disease diagnosis,^10,12^ with mixed effects on racial and socioeconomic disparities.^11,15,16,17^ The effect of Medicaid expansion on the health outcomes of patients with cancer remains unknown. Because time to treatment initiation (TTI) is associated with survival across a variety of malignant neoplasms,^18^ it may represent a leading indicator of the early effect of Medicaid expansion on health outcomes. Medicaid expansion might improve TTI through enhanced detection, earlier diagnosis, and improved access to care. On the other hand, given an already strained oncology workforce,^19,20^ Medicaid expansion might lengthen wait times and exacerbate TTI for patients with cancer. Because such patients are particularly vulnerable to treatment delays, an understanding of this potential unintended consequence is critical for policy makers weighing the merits of the ACA’s Medicaid expansion. The objectives of this study were to examine changes in insurance status, stage at diagnosis, and timely treatment associated with Medicaid expansion among patients with incident breast, colon, and lung cancer.

## Methods

### Study Design

We conducted a quasi-experimental, difference-in-differences (DID) cross-sectional analysis comparing insurance status, cancer stage at diagnosis, and timely treatment among patients residing in Medicaid expansion and nonexpansion states before and after ACA implementation. Given that 24 states and the District of Columbia expanded their Medicaid programs on January 1, 2014, this date marked the beginning of the postexpansion period in our main analysis, which included data from January 1, 2011, to December 31, 2016. The study adhered to the Strengthening the Reporting of Observational Studies in Epidemiology (STROBE) reporting guideline.^21^ The University of Pennsylvania exempted the study from institutional review board approval and informed consent because it involved deidentified patient data only.

### Data Source

Data were obtained from the National Cancer Database (NCDB), a hospital-based registry jointly sponsored by the American College of Surgeons and American Cancer Society. The NCDB contains patient-level data on all incident cancer diagnoses from more than 1500 member institutions, representing more than 70% of all new cancer diagnoses in the United States from approximately 30% of all US hospitals.^22,23^ Data collection is standardized based on the Facility Oncology Registry Data Standards^24^ and includes registrar-abstracted data on patient, tumor, treatment, and hospital characteristics.

### Population

We assembled cohorts of adult nonelderly patients with newly diagnosed breast (*International Classification of Diseases for Oncology, Third Edition* [*ICD-O-3*] codes C50.0-50.9), colon (*ICD-O-3* codes C18.0-18.9), and non–small cell lung (*ICD-O-3* codes C34.0-34.9, excluding histology codes 8041-8045) cancer from 2011 to 2016. We selected these cancers because they are common, amenable to screening, and often treated with curative intent in the nonmetastatic setting, and thus outcomes for patients with these cancers might be particularly sensitive to changes in insurance and access to care. The NCDB includes a variable indicating the expansion status of the patient’s state of residence for patients 40 years and older. Patients younger than 40 years, for whom expansion status was unavailable, were excluded. In addition, we excluded patients with noninvasive in situ cancers, rare clinical presentations (eg, male breast cancer), and rare histology.

We identified 963 509 eligible patients aged 40 to 64 years with a new diagnosis of invasive breast, colon, or non–small cell lung cancer during the study period. We then excluded patients with missing data on insurance status (18 651 [1.9%]) and cancer stage (19 315 [2.0%]). The final cohort for our initial analyses on insurance status and stage included 925 543 patients. A total of 881 241 patients survived at least 30 days after diagnosis and received treatment within 365 days and were therefore eligible for subsequent analyses on timely treatment. Patients with missing or unknown treatment data were excluded (32 912 [3.7%]). The final cohort for treatment analyses included 848 329 patients. eFigure 1 in the Supplement depicts the study population flowchart.

### Measures

The primary outcomes were insurance status, stage, and timely treatment within 30 and 90 days of diagnosis. Insurance status and stage were defined by preexisting variables in the NCDB indicating insurance status and American Joint Committee on Cancer (AJCC) stage at diagnosis, respectively. From these variables, we derived patient-level binary variables indicating whether patients had no insurance, Medicaid insurance, early-stage cancer (AJCC stage I), and advanced-stage cancer (AJCC stage IV) at diagnosis.

For our main analysis, TTI was defined as days from diagnosis to the earliest cancer-directed treatment of any type and dichotomized into 2 patient-centered, clinically relevant metrics: TTI within 30 and TTI within 90 days of diagnosis. Strong data support the clinical benefit of a short TTI in nonmetastatic breast cancer^25,26,27,28,29^; the optimal TTI for non–small cell lung cancer and colon cancer are presumed to be short but less well defined.^30,31,32,33,34,35,36^ The NCDB records the date of diagnosis as that of the most definitive diagnostic confirmation based on histologic, cytologic, or immunohistochemical findings from biopsy specimens in the patient’s record. Time to treatment is coded in distinct variables delineating time to first surgery, most definitive surgery (eg, lumpectomy followed by mastectomy, time from diagnosis to mastectomy), radiotherapy, and systemic therapy (eg, chemotherapy, immunotherapy, or hormonal therapy). From these, we derived a variable indicating the number of days from diagnosis to first cancer-directed therapy.

In prespecified subgroup analyses, TTI was alternatively defined as time to curative-intent surgery among those with nonmetastatic disease undergoing surgical treatment (n = 650 700) and as time to palliative-intent systemic therapy among those with metastatic disease undergoing systemic therapy (n = 117 877). For those with nonmetastatic disease, an indicator variable available in the NCDB distinguished curative treatment from the same modality used strictly for palliation. For those with metastatic disease, systemic treatment was assumed to be palliative. Data were available for the first course of treatment only.

The primary independent variable was an interaction between residence in a Medicaid expansion state and cancer diagnosis in the postexpansion period (2014-2016). Based on prior literature,^18,25,26,27,28,29,30,31,32,33,34,35,36^ patient-level covariates included age, sex, race/ethnicity, insurance status, income, educational level, rurality, distance to hospital facility, hospital transfer, Charlson-Deyo comorbidity,^37^ multiple malignant neoplasms, cancer type, and stage at diagnosis. The NCDB estimates median family income and educational attainment using data from the 2016 American Community Survey linked with the patient’s zip code of residence at diagnosis. Hospital-level covariates included facility type (ie, community vs academic) and geographic region.

### Statistical Analysis

**Figure 1.**
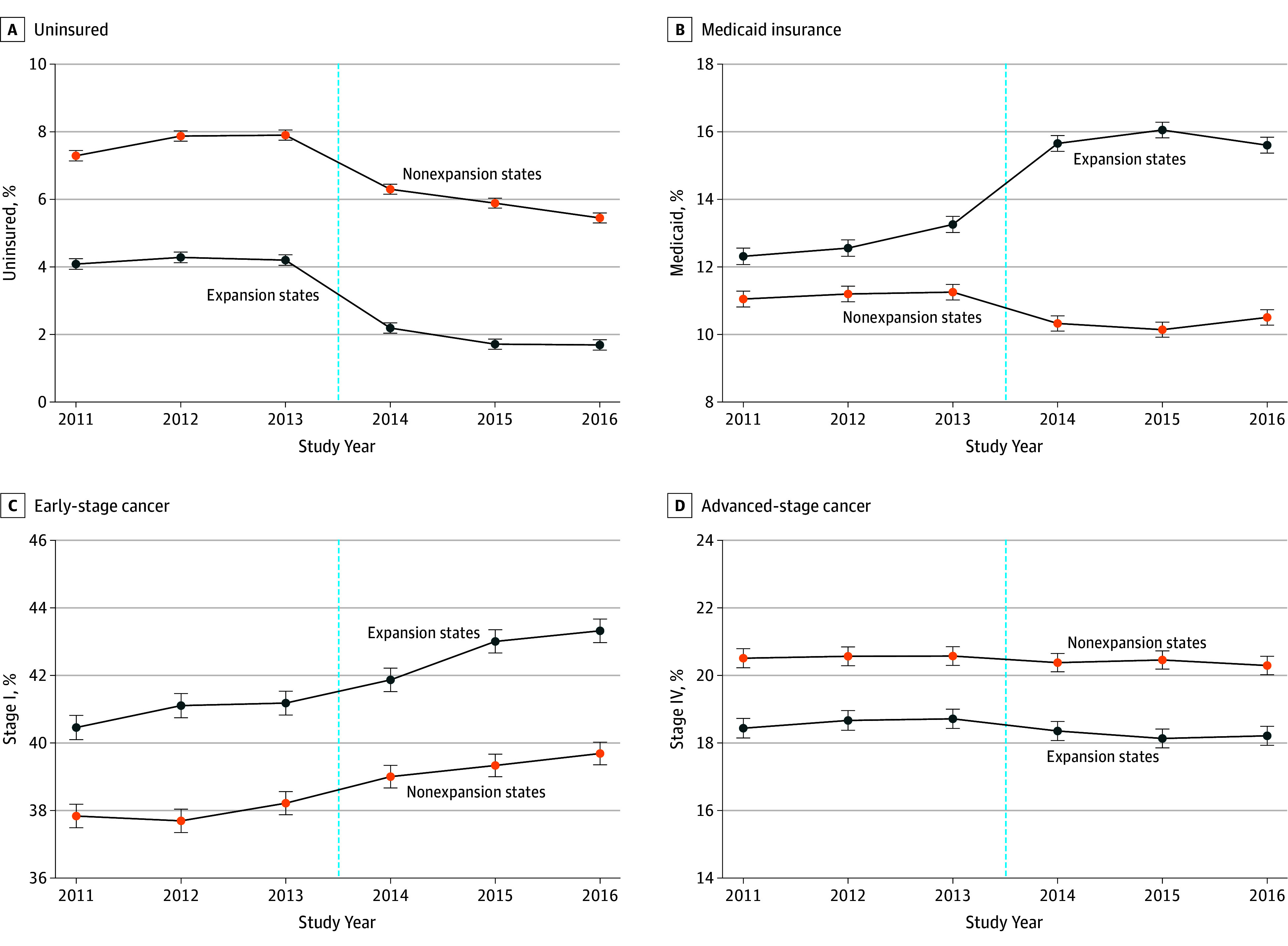
Unadjusted Trends in Health Insurance Status and Cancer Stage at Diagnosis by State Medicaid Expansion Status Participants included patients aged 40 to 64 years with incident breast, colon, and lung cancer in the National Cancer Database from January 1, 2011, to December 31, 2016. Error bars represent 95% CIs of estimated margins. The vertical dashed line represents January 1, 2014, the date of Medicaid expansion.

**Figure 2.**
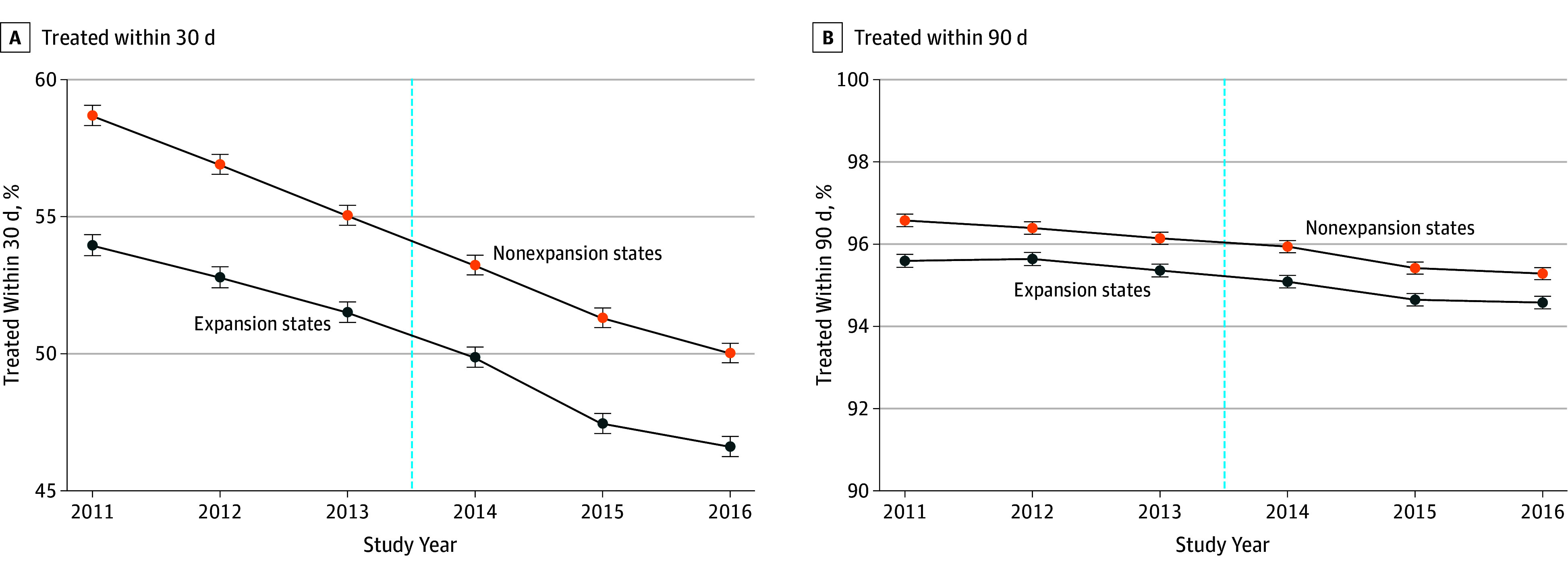
Unadjusted Trends in Timely Treatment by State Medicaid Expansion Status Participants included patients aged 40 to 64 years with incident breast, colon, and lung cancer in the National Cancer Database from January 1, 2011, to December 31, 2016. Error bars represent 95% CIs of estimated margins. The vertical dashed line represents January 1, 2014, the date of Medicaid expansion.

A primary assumption in the DID approach is parallel trends in the preexposure period. We tested this assumption graphically (Figures 1 and 2) and by conducting a falsification test regressing a linear year-by-expansion interaction on each outcome in the preexpansion period only (eTable 1 in the Supplement). Nonsignificant interactions in the preexpansion period confirmed that trends in outcomes did not differ significantly between expansion and nonexpansion states before 2014 (using a Bonferroni-corrected significance threshold of 2-sided *P* < .008) except in the case of percentage of Medicaid-insured patients, for which divergent trends began in the preexpansion period.

We then calculated DID estimates using multivariable linear regression to estimate each outcome as a function of residing in an expansion state, diagnosis in the postexpansion period, and an interaction between the two. We elected to use linear models because they are commonly used in DID analyses and provide straightforward estimates of absolute changes.^38,39^ All outcomes were modeled as dichotomous variables, with mean estimates representing the proportion of patients with a particular insurance status, diagnosis stage, or treatment interval. In the model for Medicaid insurance, we additionally adjusted for preexpansion time trends given the lack of parallel trends for this outcome. In all models, we calculated robust standard errors accounting for clustering within the hospital and included the covariates defined above and hospital-level fixed effects.

### Sensitivity Analyses

We tested the sensitivity of key findings to alternative populations and model specifications. First, we excluded patients residing in states that expanded their Medicaid programs within the study period but before (California; Connecticut; Washington, DC; Minnesota; New Jersey; and Washington) or after (Mississippi, New Hampshire, Pennsylvania, Indiana, Arkansas, Montana, and Louisiana) January 1, 2014, because inclusion of these groups might be expected to dilute the measurable effect of expansions that occurred on January 1, 2014. Second, we excluded patients with a TTI of zero (52 130 [5.9%]), indicating extirpation of the primary tumor at the time of diagnosis, because Medicaid expansion might not be expected to affect the interval between diagnosis and treatment for these patients, whose inclusion might therefore dilute an observable effect. Third, we adjusted for preexpansion linear time trends in models for outcomes with nonparallel trends using a significance threshold of 2-sided *P* < .05 (ie, percentage uninsured and percentage with TTI <30 days).

Data were analyzed from March 8 to August 15, 2019, using Stata software, version 15 (StataCorp LLC). All analyses used 2-tailed testing with a significance threshold of *P* < .008 (Bonferroni correction for testing 6 hypotheses).

## Results

### Study Population

The study population included 925 543 patients (21.4% men and 78.6% women; mean [SD] age, 55.0 [6.5] years; 14.2% black and 5.7% Hispanic) with a new diagnosis of invasive breast (58.9%), colon (14.6%), or non–small cell lung (26.5%) cancer. In all, 446 888 (48.3%) resided in expansion states (mean [SD] age, 54.9 [6.5] years; 80.0% women and 20.0% men; 10.9% black; and 6.8% Hispanic) and 478 655 (51.7%) resided in nonexpansion states (mean [SD] age, 55.1 [6.5] years; 77.3% women and 22.7% men; 17.4% black; and 4.7% Hispanic) (Table 1). Those living in expansion states were more likely to reside in wealthier (47.4% vs 26.9%) and metropolitan (85.9% vs 80.5%) zip codes, have no comorbidities (80.0% vs 77.1%), and seek cancer care at an academic center (41.1% vs 28.4%) and were less likely to be uninsured (3.0% vs 6.8%) or present with metastatic cancer (18.4% vs 20.5%).

**Table 1.** Characteristics of Patients With Incident Breast, Colon, and Lung Cancer by State Medicaid Expansion Status^a^

Characteristic	State Medicaid Expansion Status^b^		
	Nonexpansion (n = 478 655)	Expansion (n = 446 888)	All (N = 925 543)
Age, mean (SD), y	55.1 (6.5)	54.9 (6.5)	55.0 (6.5)
Sex			
Male	108 773 (22.7)	89 361 (20.0)	198 134 (21.4)
Female	369 882 (77.3)	357 527 (80.0)	727 409 (78.6)
Race			
White	378 661 (79.1)	360 197 (80.6)	738 858 (79.8)
Black	83 116 (17.4)	48 685 (10.9)	131 801 (14.2)
Asian and Pacific Islander	8410 (1.8)	26 889 (6.0)	35 299 (3.8)
Other	5329 (1.1)	6492 (1.5)	11 821 (1.3)
Missing	3139 (0.7)	4625 (1.0)	7764 (0.8)
Ethnicity			
Non-Hispanic	442 221 (92.4)	405 671 (90.8)	847 892 (91.6)
Hispanic	22 414 (4.7)	30 431 (6.8)	52 845 (5.7)
Missing	14 020 (2.9)	10 786 (2.4)	24 806 (2.7)
Insurance			
Uninsured	32 341 (6.8)	13 369 (3.0)	45 710 (4.9)
Private	327 218 (68.4)	319 834 (71.6)	647 052 (69.9)
Medicaid	51 377 (10.7)	63 844 (14.3)	115 221 (12.4)
Medicare	56 637 (11.8)	44 779 (10.0)	101 416 (11.0)
Other government	11 082 (2.3)	5062 (1.1)	16 144 (1.7)
Income quartile by zip code, US$^c^			
<38 000	112 891 (23.6)	58 554 (13.1)	171 445 (18.5)
38 000-47 999	120 044 (25.1)	73 574 (16.5)	193 618 (20.9)
48 000-62 999	110 429 (23.1)	97 646 (21.9)	208 075 (22.5)
≥63 000	128 657 (26.9)	211 824 (47.4)	340 481 (36.8)
Missing	6634 (1.4)	5290 (1.2)	11 924 (1.3)
Educational level by zip code quartile, % not graduating high school^d^			
>17.5	110 295 (23.0)	83 173 (18.6)	193 468 (20.9)
10.9-17.5	134 622 (28.1)	101 774 (22.8)	236 396 (25.5)
6.3-10.8	127 847 (26.7)	127 694 (28.6)	255 541 (27.6)
<6.3	99 979 (20.9)	129 821 (29.1)	229 800 (24.8)
Missing	5912 (1.2)	4426 (1.0)	10 338 (1.1)
Rurality^e^			
Metropolitan	385 155 (80.5)	384 011 (85.9)	769 166 (83.1)
Urban	73 037 (15.3)	45 536 (10.2)	118 573 (12.8)
Rural	10 112 (2.1)	5095 (1.1)	15 207 (1.6)
Missing	10 351 (2.2)	12 246 (2.7)	22 597 (2.4)
Charlson-Deyo comorbidity score			
0	369 146 (77.1)	357 610 (80.0)	726 756 (78.5)
1	81 450 (17.0)	66 807 (14.9)	148 257 (16.0)
2	19 622 (4.1)	15 586 (3.5)	35 208 (3.8)
≥3	8437 (1.8)	6885 (1.5)	15 322 (1.7)
Cancer type			
Breast	273 476 (57.1)	272 119 (60.9)	545 595 (58.9)
Colon	71 799 (15.0)	62 970 (14.1)	134 769 (14.6)
Lung	133 380 (27.9)	111 799 (25.0)	245 179 (26.5)
AJCC stage			
I	185 003 (38.7)	187 023 (41.9)	372 026 (40.2)
II	117 404 (24.5)	109 483 (24.5)	226 887 (24.5)
III	78 321 (16.4)	68 095 (15.2)	146 416 (15.8)
IV	97 927 (20.5)	82 287 (18.4)	180 214 (19.5)
Facility type			
Community	342 944 (71.6)	263 020 (58.9)	605 964 (65.5)
Academic	135 711 (28.4)	183 868 (41.1)	319 579 (34.5)

Abbreviation: AJCC, American Joint Committee on Cancer.

^a^ Participants include patients aged 40 to 64 years in the National Cancer Database from January 1, 2011, to December 31, 2016.

^b^ Unless otherwise indicated, data are expressed as number (percentage) of patients. Percentages are rounded and may not total 100.

^c^ Estimated by matching the zip code of the patient recorded at the time of diagnosis against 2016 American Community Survey data, spanning years 2012 to 2016 and adjusted for 2016 inflation. Household income is categorized as quartiles based on equally proportioned income ranges among all US zip codes.

^d^ Estimated by matching the zip code of the patient at diagnosis to 2016 American Community Survey data, which span the years 2012 to 2016. This item provides a measure of the number of adults 25 years or older in the patient’s zip code who did not graduate from high school and is categorized as equally proportioned quartiles among all US zip codes.

^e^ Estimated by matching the state/county Federal Information Processing Standards code of the patient at diagnosis to 2013 data published by the US Department of Agriculture Economic Research Service. Metropolitan counties are defined as having a population size of the metropolitan area greater than 250 000. Urban counties are defined as nonmetropolitan with a population size of at least 2500. Rural counties have a population of fewer than 2500.

### Insurance Status

Figure 1A and B illustrate unadjusted trends in insurance coverage at diagnosis by state expansion status. Expansion states had lower rates of uninsured patients throughout the study period. Although the percentage of uninsured patients declined in both groups after Medicaid expansion, decreases were significantly greater in expansion compared with nonexpansion states. Table 2 shows unadjusted and adjusted DID estimates for insurance coverage at diagnosis. In Medicaid expansion states compared with nonexpansion states, the percentage of uninsured decreased (adjusted DID, −0.7 [95% CI, −1.2 to −0.3] percentage points; *P* = .001), and the percentage of Medicaid-insured increased (adjusted DID, 3.3 [95% CI, 2.5 to 4.1] percentage points; *P* < .001) after expansion. The estimates were similar with and without multivariable adjustment.

**Table 2.** Changes in Insurance Status and Stage at Diagnosis After Medicaid Expansion Among Patients With Incident Breast, Colon, and Lung Cancer^a^

Outcome	State Medicaid Status						Adjusted DID (95% CI), Percentage Points^b^	*P* Value
	Expansion			Nonexpansion				
	Unadjusted % of Patients		Unadjusted Difference, Percentage Points	Unadjusted % of Patients		Unadjusted Difference, Percentage Points		
	Before	After		Before	After			
Insurance status								
Uninsured	4.2	1.9	−2.3 (−2.5 to −2.2)	7.7	5.9	−1.8 (−1.9 to −1.7)	−0.7 (−1.2 to −0.3)	.001
Medicaid	12.7	15.8	3.1 (2.9 to 3.3)	11.2	10.3	−0.9 (−1.0 to −0.7)	3.3 (2.5 to 4.1)	<.001
AJCC stage at diagnosis								
I	40.9	42.7	1.8 (1.5 to 2.1)	37.9	39.3	1.4 (1.2 to 1.7)	0.8 (0.3 to 1.2)	.001
IV	18.6	18.2	−0.4 (−0.6 to −0.1)	20.5	20.4	−0.1 (−0.4 to 0.0)	−0.5 (−0.9 to −0.2)	.003

Abbreviations: AJCC, American Joint Committee on Cancer; DID, difference-in-differences.

^a^ Participants include patients aged 40 to 64 years in the National Cancer Database from January 1, 2011, to December 31, 2016.

^b^ Indicates the regression coefficient on an interaction term between residence in an expansion state and diagnosis in the postexpansion period, adjusted for patient age, sex, race, ethnicity, income, insurance status (stage analyses only), educational level, rurality, comorbidity, multiple malignant neoplasms, hospital transfer, primary site, and diagnosis stage (insurance analyses only).

### Stage at Diagnosis

Figure 1C and D illustrate unadjusted trends in cancer stage at diagnosis by state expansion status. Patients residing in expansion states were more likely to be diagnosed with early-stage cancers and less likely to be diagnosed with advanced-stage cancers throughout the study period. The percentage of early-stage cancer diagnoses increased over time in expansion and nonexpansion states, whereas the percentage of advanced-stage cancer diagnoses remained relatively constant. Table 2 shows unadjusted and adjusted DID estimates for stage at diagnosis. In Medicaid expansion states compared with nonexpansion states, a small but statistically significant increase occurred in the percentage of early-stage cancer diagnosis (adjusted DID, 0.8 [95% CI, 0.3 to 1.2] percentage points; *P* = .001), and a decrease occurred in the percentage of advanced-stage cancer diagnoses (adjusted DID, −0.5 [95% CI, −0.9 to −0.2] percentage points; *P* = .003) after expansion.

### Timely Treatment

Figure 2 and Table 3 illustrate trends in the percentage of patients receiving timely treatment within 30 and 90 days of diagnosis among those receiving cancer-directed treatment. The percentages treated within 30 and 90 days were greater in nonexpansion states and decreased over time throughout the study period for both groups. In Medicaid expansion states, the percentage treated within 30 days declined from 52.7% before expansion to 48.0% after expansion (difference, −4.7 [95% CI, −5.1 to −4.5] percentage points). In nonexpansion states, this percentage declined from 56.9% before expansion to 51.5% after expansion (difference, −5.4 [95% CI, −5.6 to −5.1] percentage points), yielding no statistically significant DID in timely treatment by state expansion status (adjusted DID, 0.6 [95% CI, −0.2 to 1.4] percentage points; *P* = .14). Results were similarly nonsignificant when analyzing the percentage treated within 90 days of diagnosis.

**Table 3.** Changes in Timely Treatment After Medicaid Expansion Among Patients With Incident Breast, Colon, and Lung Cancer^a^

Outcome	State Medicaid Status						Adjusted DID (95% CI), Percentage Points^b^	*P* Value
	Expansion			Nonexpansion				
	Unadjusted % of Patients		Unadjusted Difference, Percentage Points	Unadjusted % of Patients		Unadjusted Difference, Percentage Points		
	Before	After		Before	After			
**All Treated Patients, Time to First Treatment**								
TTI <30 d	52.7	48.0	−4.7 (−5.1 to −4.5)	56.9	51.5	−5.4 (−5.6 to −5.1)	0.6 (−0.2 to 1.4)	.14
TTI <90 d	95.5	94.8	−0.7 (−0.9 to −0.6)	96.4	95.5	−0.9 (−0.9 to −0.7)	0.2 (−0.1 to 0.4)	.18
**Patients Treated for Nonmetastatic Cancer, Time to Surgery**								
TTI <30 d	50.8	45.0	−5.8 (−6.2 to −5.5)	54.1	47.8	−6.3 (−6.7 to −6.0)	0.2 (−0.7 to 1.1)	.67
TTI <90 d	89.1	86.4	−2.7 (−2.9 to −2.5)	89.0	86.0	−3.0 (−3.3 to −2.8)	0.1 (−0.4 to 0.6)	.69
**Patients Treated for Metastatic Cancer, Time to Systemic Therapy**								
TTI <30 d	39.6	38.0	−1.6 (−2.5 to −0.8)	40.5	37.7	−2.8 (−3.6 to −2.1)	1.5 (0.2 to 2.7)	.03
TTI <90 d	92.1	92.5	0.4 (0.0 to 0.9)	92.6	92.2	−0.4 (−0.7 to 0.0)	1.0 (0.4 to 1.6)	.002

Abbreviations: DID, difference-in-differences; TTI, time to treatment initiation.

^a^ Participants include patients aged 40 to 64 years in the National Cancer Database from January 1, 2011, to December 31, 2016. Treatment is defined as any cancer-directed therapy, including extirpative surgical procedure to the primary site, radiotherapy, and systemic therapy (chemotherapy, immunotherapy, or hormone therapy).

^b^ Indicates the regression coefficient on an interaction term between residence in an expansion state and diagnosis in the postexpansion period, adjusted for patient age, sex, race, ethnicity, insurance status, income, educational level, rurality, comorbidity, multiple malignant neoplasms, hospital transfer, primary site, and diagnosis stage.

Among patients with nonmetastatic cancer who underwent curative-intent surgery, the percentage treated within 30 and 90 days of diagnosis declined in expansion and nonexpansion states across the study period (eFigure 2 in the Supplement and Table 3). The differences in decline were not statistically significant (adjusted DID for TTI <30 days, 0.2 [95% CI, −0.7 to 1.1] percentage points [*P* = .67]; adjusted DID for TTI <90 days, 0.1 [95% CI, −0.4 to 0.6] percentage points [*P* = .69]). Among patients with metastatic cancer who received systemic therapy, a statistically significant increase after expansion was observed in the percentage treated within 90 days in expansion compared with nonexpansion states (adjusted DID for TTI <30 days, 1.5 [95% CI, 0.2 to 2.7] percentage points [*P* = .03]; adjusted DID for TTI <90 days, 1.0 [95% CI, 0.4 to 1.6] percentage points [*P* = .002]).

### Sensitivity Analyses

eTable 2 in the Supplement presents the results of sensitivity analyses on all outcomes after excluding early and late adopters of Medicaid expansion. These analyses revealed heightened effect estimates across all outcomes, including a greater reduction in the percentage uninsured (adjusted DID, −1.0 [95% CI, −1.6 to −0.5] percentage points; *P* < .001), greater increase in percentage of early-stage cancer diagnosis (adjusted DID, 1.2 [95% CI, 0.3 to 1.2] percentage points; *P* < .001), and greater increase in timely treatment (adjusted DID for TTI <30 days, 1.3 [95% CI, 0.3 to 2.2] percentage points; *P* = .009) associated with Medicaid expansion. Treatment outcomes were also robust to the exclusion of patients with a TTI of zero (eTable 3 in the Supplement). Finally, adjustment for preexpansion time trends for percentage uninsured and percentage with TTI of less than 30 days did not affect the direction or significance of effect estimates.

## Discussion

Using a hospital-based cancer registry encompassing nearly 1 million nonelderly adults with incident breast, colon, and non–small cell lung cancer from 2011 to 2016, we found that the ACA’s Medicaid expansion was associated with a decreased rate of uninsured patients and a shift toward earlier-stage cancer diagnosis and was not associated with changes in the timeliness of treatment initiation. These key findings corroborate and extend those of earlier studies,^10,11,12^ in which follow-up time to examine the effect of expansions on insurance status and stage at diagnosis was limited to only 1 year after ACA implementation. Because lack of insurance is associated with poor health outcomes and because stage is one of the most potent factors associated with survival for patients with cancer,^40,41,42^ our findings suggest definitive and persistent benefit 3 years after ACA implementation to nonelderly patients newly diagnosed with breast, colon, and non–small cell lung cancer residing in states that adopted Medicaid expansion.

In addition, we found no association of Medicaid expansion with the timeliness of treatment initiation. For patients with cancer, insurance coverage has been shown to afford better access to effective cancer therapies,^41^ which could conceivably improve rates of timely treatment in expansion states. However, if a surge in insured patients with cancer overwhelmed an already strained oncology workforce, lengthened wait times and costly treatment delays could result, as have occurred outside oncology.^2,6^ Lending further support to this hypothesis, we uncovered a temporal association with lengthened time to treatment in both expansion and nonexpansion states. This paradoxical finding warrants further study and may reflect an increasingly strained oncology workforce in the face of growing cancer prevalence, increasing complexity of care, and looming workforce shortages.^19,20,43^ In this context, it is reassuring that our study found no evidence of treatment delays associated with Medicaid expansion.

Although timeliness of care is an important patient-centered quality metric across the cancer care continuum, it is especially vital for patients with nonmetastatic disease receiving curative-intent surgery and for patients with metastatic disease receiving systemic therapy, for whom treatment delays can lead to missed opportunities for cure and life-prolonging therapy, respectively. Our analyses of these prespecified subgroups revealed no evidence for differential rates of timely treatment by state Medicaid expansion status among patients with nonmetastatic disease undergoing curative surgical treatment and a small, statistically significant increase in the proportion of patients with metastatic disease receiving timely systemic therapy within 90 days of diagnosis in expansion states. However, given the small absolute effect size noted in a subpopulation only, this result should be interpreted cautiously.

Sensitivity analyses excluding early and late adopters of Medicaid expansion yielded greater effect estimates across all outcomes, including a small improvement in timely treatment associated with Medicaid expansion. This outcome underscores the hypothesis that the inclusion of these groups in our main analysis might have diluted the measurable effect of the expansions that occurred on January 1, 2014. Furthermore, these results suggest that our estimates of the effect of Medicaid expansion on insurance status and stage may represent underestimates of the true effect and, importantly, that a trend toward improvement in timely treatment may be associated with Medicaid expansion, which we failed to detect in our main analysis. For this reason, we believe that cancer treatment patterns associated with Medicaid expansion warrant further study.

### Limitations

This study has several limitations. First, it is observational and thus limited in its ability to prove causality. Although the quasi-experimental DID approach is commonly used in policy analyses and mitigates the effect of unmeasured confounders, including secular time trends,^44^ a possibility of omitted variable bias remains. Second, state of residence is unobserved in our data set, and consequently we did not adjust for state-level effects. However, the incorporation of hospital fixed effects may capture state-level correlation, as suggested by the fact that our results were robust to the exclusion of early and late adopters of Medicaid expansion. Third, the NCDB is a hospital-based rather than population-based cancer registry. However, the NCDB has significant penetration into both community and academic sites across urban and rural areas,^23^ and its demographics have been shown to be similar to those of the Surveillance, Epidemiology, and End Results (SEER) registries.^45^ Fourth, our study is limited to patients aged 40 to 64 years with breast, colon, and non–small cell lung cancer and to evaluating state Medicaid expansions occurring on January 1, 2014. Future studies encompassing other tumor types, a broader age range, and more contemporary data sets are necessary to evaluate the generalizability of our findings.

## Conclusions

Among patients with newly diagnosed breast, colon, and non–small cell lung cancer, the ACA’s Medicaid expansion was associated with a decreased rate of uninsured patients and an increased rate of early-stage cancer diagnosis. For patients initiating cancer-directed therapy, no association was found between Medicaid expansion and differential rates of timely treatment, suggesting neither an improvement nor a decrement in the timeliness of cancer care delivery. Further research is needed to understand Medicaid expansion’s effect on the treatment patterns and health outcomes of patients with cancer.

## Notes

Supplement. **eFigure 1. **Study Population Flowchart **eFigure 2. **Unadjusted Trends in Timely Treatment by State Medicaid Expansion Status, Among Subgroups **eTable 1. **Test of Parallel Trends: Falsification Test of Year-by-Expansion Interaction in Preexpansion Period **eTable 2. **Sensitivity Analyses of Insurance Status, Stage, and Timely Treatment, After Excluding Early and Late Adopters of Medicaid Expansion **eTable 3. **Sensitivity Analyses of Timely Treatment, After Excluding Patients With Time-to-Treatment of Zero
