# Supplementary material for: Association of Medicaid Expansion Under the Affordable Care Act With Insurance Status, Cancer Stage, and Timely Treatment Among Patients With Breast, Colon, and Lung Cancer
Source: JAMA Netw Open. 2020 Feb 19;3(2):e1921653. doi: 10.1001/jamanetworkopen.2019.21653 (PMC12549135; doi:10.1001/jamanetworkopen.2019.21653)
Supplement: Supplement. — eFigure 1. Study Population Flowchart eFigure 2. Unadjusted Trends in Timely Treatment by State Medicaid Expansion Status, Among Subgroups eTable 1. Test of Parallel Trends: Falsification Test of Year-by-Expansion Interaction in Preexpansion Period eTable 2. Sensitivity Analyses of Insurance Status, Stage, and Timely Treatment, After Excluding Early and Late Adopters of Medicaid Expansion eTable 3. Sensitivity Analyses of Timely Treatment, After Excluding Patients With Time-to-Treatment of Zero [file jamanetwopen-e1921653-s001.pdf]

# Supplementary Online Content

Takvorian SU, Oganisian A, Mamtani R, et al. Association of Medicaid expansion under the Affordable Care Act with insurance status, cancer stage, and timely treatment among patients with breast, colon, and lung cancer. *JAMA Netw Open*. 2020;3(2):e1921653. doi:10.1001/jamanetworkopen.2019.21653

**eFigure 1.** Study Population Flowchart

**eFigure 2.** Unadjusted Trends in Timely Treatment by State Medicaid Expansion Status, Among Subgroups

**eTable 1.** Test of Parallel Trends: Falsification Test of Year-by-Expansion Interaction in Preexpansion Period

**eTable 2.** Sensitivity Analyses of Insurance Status, Stage, and Timely Treatment, After Excluding Early and Late Adopters of Medicaid Expansion

**eTable 3.** Sensitivity Analyses of Timely Treatment, After Excluding Patients With Time-to-Treatment of Zero

This supplementary material has been provided by the authors to give readers additional information about their work.

©2020 Takvorian SU et al. *JAMA Network Open*.

## eFigure 1. Study Population Flowchart

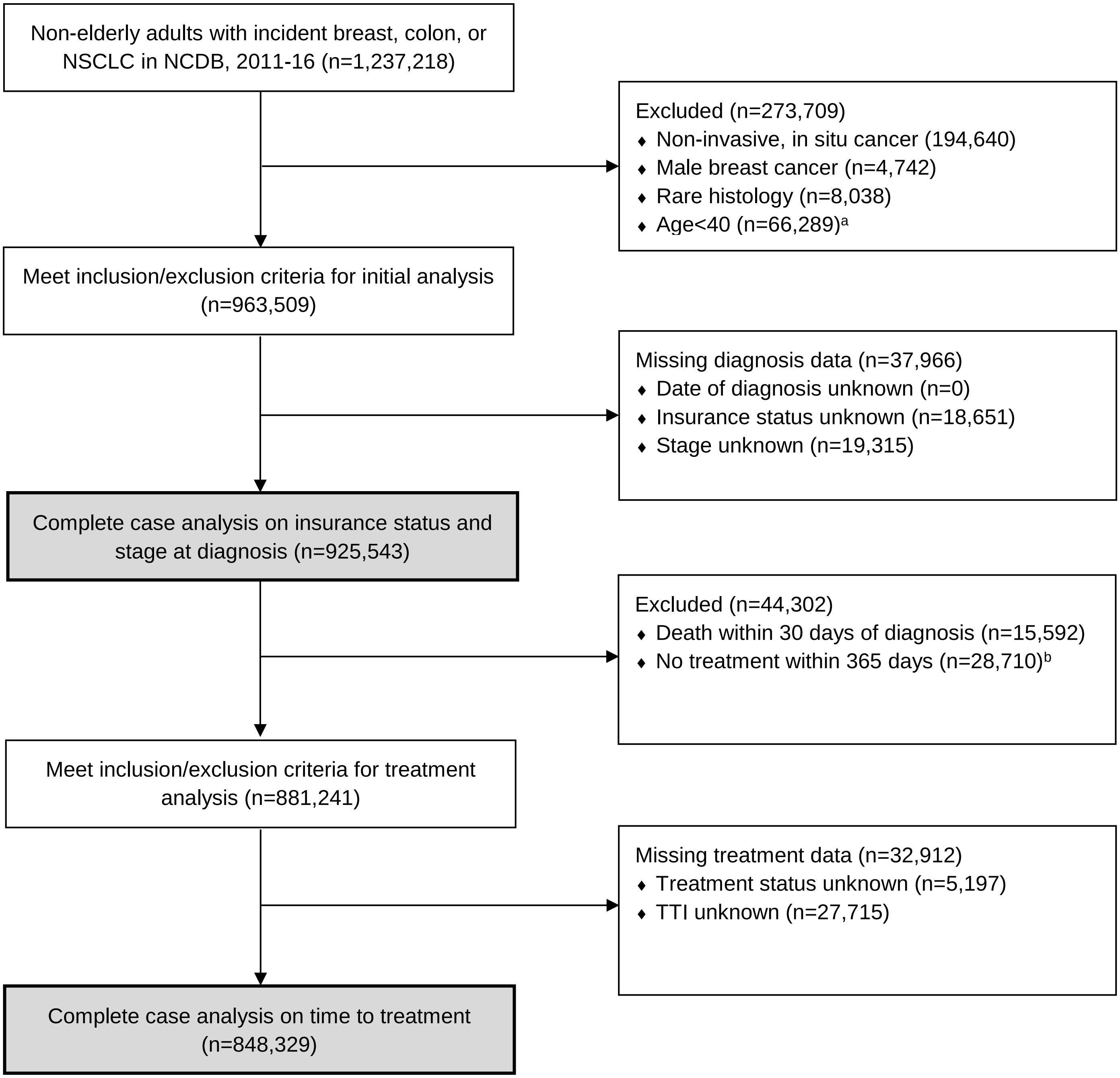


NSCLC= Non-small cell lung cancer
NCDB= National Cancer Database
TTI= Time to treatment initiation

[a] State of residence and therefore expansion status is suppressed for patients age<40 in National Cancer Database.

[b] Treatment is defined as cancer-directed therapy, including extirpative surgical procedure to the primary site, radiation therapy, and systemic therapy (chemotherapy, immunotherapy, hormone therapy).

©2020 Takvorian SU et al. *JAMA Network Open*.

## eFigure 2. Unadjusted Trends in Timely Treatment by State Medicaid Expansion Status, Among Subgroups

*a) Percent treated with surgery within 30 days of diagnosis, among non-metastatic patients*

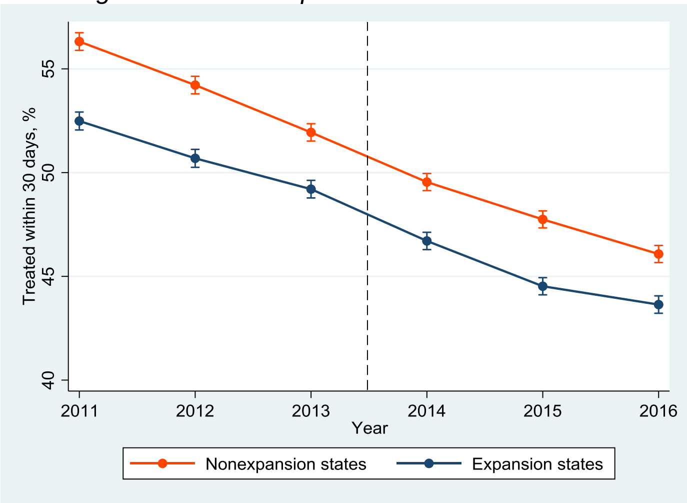


*b) Percent treated with surgery within 90 days of diagnosis, among non-metastatic patients*

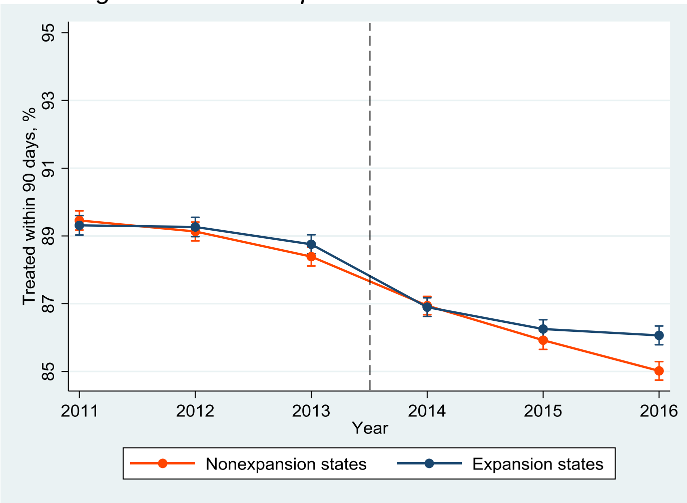


*c) Percent treated with systemic therapy within 30 days of diagnosis, among metastatic patients*

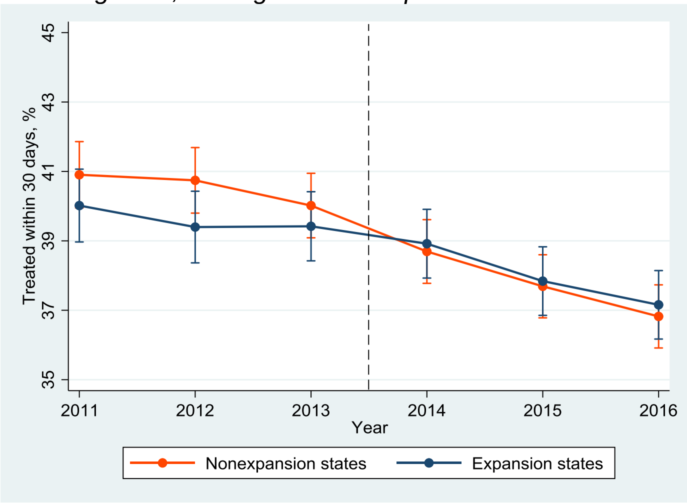


*d) Percent treated with systemic therapy within 90 days of diagnosis, among metastatic patients*

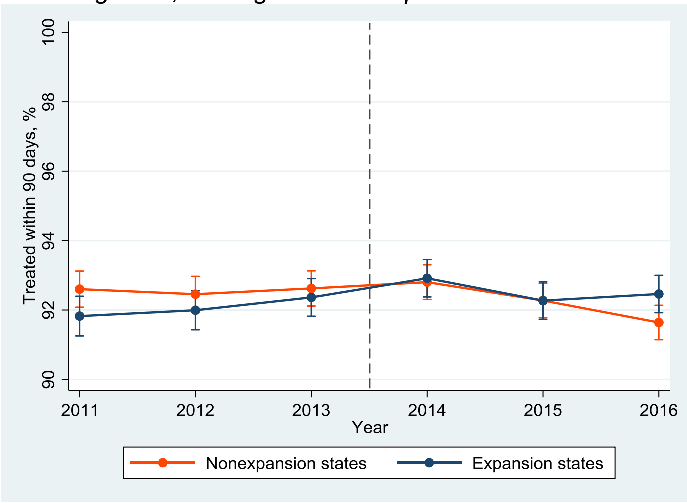


Solid lines connect unadjusted annual rates of timely treatment among subgroups, by Medicaid expansion status. Error bars represent 95% confidence intervals of predicted margins. The vertical hashed line represents January 1, 2014 as the date of Medicaid expansion.

©2020 Takvorian SU et al. *JAMA Network Open*.

**eTable 1. Test of Parallel Trends: Falsification Test of Year-by-Expansion Interaction in Preexpansion Period**

| Outcome | Adjusted DiD[a], percentage points (95% CI) | p-value |
|---|---|---|
| *Insurance status* | | |
| Uninsured | -0.3 (-0.5 to -0.1) | 0.01 |
| Medicaid | +0.5 (+0.2 to +0.8) | 0.004[b] |
| *Diagnosis stage* | | |
| Stage I | +0.2 (-0.2 to +0.5) | 0.27 |
| Stage IV | +0.1 (-0.1 to +0.4) | 0.35 |
| *Timely treatment (all treated patients, time to any treatment)* | | |
| TTI[c]<30 days | +0.5 (+0.0 to +1.0) | 0.03 |
| TTI<90 days | +0.1 (-0.1 to +0.3) | 0.22 |
| *Timely treatment (non-metastatic patients, time to surgery)* | | |
| TTI<30 days | +0.4 (-0.1 to +1.0) | 0.12 |
| TTI<90 days | +0.2 (-0.1 to +0.5) | 0.27 |
| *Timely treatment (metastatic patients, time to systemic therapy)* | | |
| TTI<30 days | +0.2 (-0.1 to +1.2) | 0.69 |
| TTI<90 days | +0.3 (-0.3 to +0.9) | 0.27 |

[a] Adjusted difference-in-differences (DiD) estimate for each outcome is the regression coefficient on an interaction term between residence in an expansion state and a linear time trend. Data limited to pre-expansion period only.

[b] Significant year-by-expansion interaction suggests possible violation of pre-expansion parallel trends assumption.

[c] Time to treatment initiation (TTI)

©2020 Takvorian SU et al. *JAMA Network Open*.

**eTable 2. Sensitivity Analyses of Insurance Status, Stage, and Timely Treatment, After Excluding Early and Late Adopters of Medicaid Expansion**

| | Expansion states (unadjusted) | | | Nonexpansion states (unadjusted) | | | Adjusted DiD[a], percentage points (95% CI) | p-value |
|---|---|---|---|---|---|---|---|---|
| **Outcome** | **Before, %** | **After, %** | **Difference, percentage points** | **Before, %** | **After, %** | **Difference, percentage points** | | |
| *Insurance status* | | | | | | | | |
| Uninsured | 4.5 | 1.9 | -2.6 | 8.9 | 7.0 | -1.9 | -1.0 (-1.6 to -0.5) | <0.001 |
| Medicaid | 12.7 | 16.2 | +3.5 | 11.1 | 9.8 | -1.3 | +4.9 (+4.2 to +5.6) | <0.001 |
| *Diagnosis stage* | | | | | | | | |
| Stage I | 40.7 | 42.7 | +2.0 | 37.5 | 38.8 | +1.3 | +1.2 (+0.3 to +1.2) | <0.001 |
| Stage IV | 19.2 | 18.8 | -0.4 | 20.6 | 20.3 | -0.3 | -0.7 (-1.1 to -0.3) | 0.001 |
| *Timely treatment (all treated patients, time to any treatment)* | | | | | | | | |
| TTI[b]<30 days | 53.4 | 48.7 | -4.7 | 57.8 | 52.0 | -5.8 | +1.3 (+0.3 to +2.2) | 0.009 |
| TTI<90 days | 96.0 | 95.3 | -0.7 | 96.4 | 95.5 | -0.9 | +0.4 (+0.1 to +0.6) | 0.009 |
| *Timely treatment (non-metastatic patients, time to surgery)* | | | | | | | | |
| TTI<30 days | 51.1 | 45.5 | -5.6 | 54.7 | 47.8 | -6.9 | +1.0 (-0.1 to +2.2) | 0.08 |
| TTI<90 days | 89.6 | 87.2 | -2.4 | 88.5 | 85.3 | -3.2 | +0.7 (+0.1 to +1.3) | 0.02 |
| *Timely treatment (metastatic patients, time to systemic therapy)* | | | | | | | | |
| TTI<30 days | 39.0 | 37.4 | -1.6 | 41.3 | 38.5 | -2.8 | +1.7 (+0.3 to +3.2) | 0.02 |
| TTI<90 days | 92.2 | 92.7 | +0.5 | 92.8 | 92.2 | -0.6 | +1.3 (+0.4 to +1.6) | 0.001 |

[a] Adjusted difference-in-differences (DiD) estimate for each outcome is the regression coefficient on an interaction term between residence in an expansion state and diagnosis in the post-expansion period. Adjusted for patient age, sex, race, ethnicity, insurance, income, education, rurality, comorbidity, multiple malignancy, hospital transfer, primary site, and diagnosis stage.

[b] Time to treatment initiation (TTI)

©2020 Takvorian SU et al. *JAMA Network Open*.

## eTable 3. Sensitivity Analyses of Timely Treatment, After Excluding Patients With Time-to-Treatment of Zero

| | Expansion states (unadjusted) | | | Nonexpansion states (unadjusted) | | | Adjusted DiD[a], percentage points (95% CI) | p-value |
|---|---|---|---|---|---|---|---|---|
| **Outcome** | **Before, %** | **After, %** | **Difference, percentage points** | **Before, %** | **After, %** | **Difference, percentage points** | | |
| *Timely treatment (all treated patients, time to any treatment)* | | | | | | | | |
| TTI[b]<30 days | 49.6 | 44.9 | -4.7 | 53.8 | 48.3 | -5.5 | +0.8 (-0.0 to +1.6) | 0.06 |
| TTI<90 days | 95.2 | 94.5 | -0.7 | 96.1 | 95.3 | -0.8 | +0.2 (-0.1 to +0.4) | 0.14 |
| *Timely treatment (non-metastatic patients, time to surgery)* | | | | | | | | |
| TTI<30 days | 47.0 | 41.2 | -5.8 | 50.2 | 43.7 | -6.5 | +0.4 (-0.6 to +1.4) | 0.40 |
| TTI<90 days | 88.3 | 85.5 | -2.8 | 88.1 | 84.9 | -3.2 | +0.2 (-0.4 to +0.7) | 0.55 |
| *Timely treatment (metastatic patients, time to systemic therapy)* | | | | | | | | |
| TTI<30 days | 40.4 | 38.7 | -1.7 | 41.4 | 38.6 | -2.8 | +1.6 (+0.3 to +2.9) | 0.02 |
| TTI<90 days | 92.2 | 92.7 | +0.5 | 92.8 | 92.3 | -0.5 | +1.1 (+0.5 to +1.8) | 0.001 |

[a] Adjusted difference-in-differences (DiD) estimate for each outcome is the regression coefficient on an interaction term between residence in an expansion state and diagnosis in the post-expansion period. Adjusted for patient age, sex, race, ethnicity, insurance, income, education, rurality, comorbidity, multiple malignancy, hospital transfer, primary site, and diagnosis stage.

[b] Time to treatment initiation (TTI)

©2020 Takvorian SU et al. *JAMA Network Open*.
